# Significant impact of three-dimensional volumetry of perinephric fat on the console time during robot-assisted partial nephrectomy

**DOI:** 10.1186/s12894-019-0567-0

**Published:** 2019-12-12

**Authors:** Daisuke Motoyama, Yuto Matsushita, Hiromitsu Watanabe, Keita Tamura, Toshiki Ito, Takayuki Sugiyama, Atsushi Otsuka, Hideaki Miyake

**Affiliations:** Department of Urology, Hamamatsu University School of Medicine, 1-20-1 Handayama, Higashi-Ku, Hamamatsu, 431-3192 Japan

**Keywords:** Three-dimensional volumetry, Perinephric fat, Robot-assisted partial nephrectomy, Mayo adhesive probability score

## Abstract

**Background:**

To assess the impact of volumetry of perinephric fat (PNF) on the perioperative outcomes of robot-assisted partial nephrectomy (RAPN).

**Methods:**

Between 2016 and 2019, a single surgeon performed RAPN for 128 patients with clinical T1a-b renal tumors at our institution, and the 70 most recent patients were included in this study to minimize the effects of surgical experience. PNF was defined as a fatty area around the kidney within the anatomical structures, including the lateroconal fascia, fusion fascia, psoas muscle, lumbar quadrate muscle and diaphragm, and its volume was calculated based on reconstructed three-dimensional computed tomography images using the SYNAPSE VINCENT system.

**Results:**

In this series, the trifecta and MIC (margin, ischemia and complications) score system outcomes were achieved in 69 (98.6%) and 64 patients (91.4%), respectively. The median PNF volume in the 70 patients was 166.05 cm^3^, which was significantly correlated with both the body mass index (BMI) and Mayo adhesive probability (MAP) score (correlation coefficient = 0.68 and 0.74, respectively). There was no significant difference in the R.E.N.A.L. nephrometry score, PNF volume or console time during RAPN among 5 groups consisting of 14 consecutive patients. Of several factors examined, the console time was significantly affected by the sex, MAP score and PNF volume, and only the PNF volume was independently associated with the console time.

**Conclusion:**

Even if performed by an experienced robotic surgeon beyond the initial learning curve, the PNF volume may influence the console time during RAPN.

## Background

Partial nephrectomy (PN) is currently regarded as the standard of care for patients with localized small renal masses because it has been demonstrated to provide equivalent cancer control with the added benefit of preserving renal function when compared with radical nephrectomy [1]. In recent years, robotic technology has been applied to the field of PN, with significant advancements in surgical techniques and instruments. Robot-assisted PN (RAPN) has become prevalent due to marked improvements of perioperative outcomes [2–4], with a shorter learning curve than laparoscopic PN [5, 6]. However, it is well known that the surgical complexity of RAPN varies depending on a wide variety of factors [7]. Thus, it is not uncommon to encounter cases in which it is difficult to perform RAPN even by experienced robotic surgeons.

Currently, there are several image-based morphometry scoring systems that enable the quantification of relevant anatomical findings to help predict the potential complexity of PN, such as the R.E.N.A.L. nephrometry score, PADUA prediction score and centrality index (C-index) [8–10]; however, patient-specific factors are not considered by these systems. Of numerous non-tumor-specific factors, those associated with perinephric fat (PNF) are reported to affect the complexity of PN [11–14]. One such factor is the presence of adherent perinephric fat (APF), which makes it difficult to mobilize the kidney and isolate the renal tumor [11]. Recently, Davidiuk et al. advocated the Mayo adhesive probability (MAP) score in order to accurately predict the presence of APF [15], and its usefulness was confirmed in several previous studies [16, 17]. However, this score is somewhat subjective regarding the definitions of both APF and the score itself, and is not intended to directly predict the surgical difficulty of PN.

Considering these findings, we focused on the PNF volume, which may influence the complexity of RAPN, and conducted 3-dimensional (3D) volumetry for PNF on a total of 70 patients undergoing RAPN performed by a single experienced robotic surgeon in order to assess the impact of PNF volume on their perioperative outcomes.

## Methods

### Patients

The research ethics committee of our institution approved the design of this study (approval number, E15–115), and the need to obtain informed consent for involvement in this study from all of the included patients was waived because of its retrospective design. Between April 2016 and April 2019, RAPN was performed for a total of 128 consecutive patients with localized clinical T1a-b renal tumors by a single experienced robotic surgeon using the da Vinci Xi (Intuitive Surgical Inc., Sunnyvale, California, USA) at our institution. Of these 128, the 70 most recent patients who underwent RAPN under a uniform surgical procedure were included in order to minimize the effects of surgical experience.

### Evaluation

All 70 patients were preoperatively examined by contrast-enhanced computed tomography (CT) with a 64-detector row scanner, Aquilion (Toshiba Medical System, Tokyo, Japan) under the following conditions: tube voltage of 120 KV, tube currents depending on the CT-AEC, rotation time of 0.5 s/VOT, pitch factor of 0.828 and slice thickness of 0.5 or 0.6 mm. All data for the 70 patients concerning clinicopathological characteristics and perioperative findings were obtained from their medical records, and we defined the console time in this study as the time from roll-in to roll-out for the patient-cart of the robotic system. For each patient, the MAP score and R.E.N.A.L. nephrometry score were assessed according to the preoperative features on contrast-enhanced CT, as previously described [8, 15], and the severity of postoperative complications that occurred during hospitalization was evaluated using the Clavien-Dindo system [18]. Three to 5 days after RAPN, contrast-enhanced CT was routinely performed in order to precisely observe the postoperative status of the resected kidney, particularly the presence of renal pseudoaneurysm [19]. In this series, the achievement of trifecta, a widely employed key surrogate for successful PN, including RAPN, was defined as the simultaneous fulfillment of the three following factors: negative surgical margins, ischemia time ≤ 25 min and no postoperative complications corresponding to grade 3 or 4 using the Clavien-Dindo system [4]. The margin, ischemia and complications (MIC) score was also analyzed as an alternative strict surrogate based on the definition of ischemia time ≤ 20 min, in addition to negative surgical margins and no Clavien-Dindo grade 3 or 4 complications [20].

### Surgical procedure

A single trained surgeon (H.M.) performed RAPN for all 70 patients included in this study employing a 3-arm da Vinci Xi robotic system. Before RAPN, 3D images were reconstructed from the digital imaging and communications in medicine (DICOM) data of the contrast-enhanced CT images for each patient using the SYNAPSE VINCENT system (FUJIFILM, Inc., Tokyo, Japan), which were directly visualized on the screen of the surgeon’s console with the TilePro multi-input display functions during RAPN [21]. At our institution, the trans-peritoneal approach is often selected, except for patients with dorsal hilar tumors or a history of intra-peritoneal surgery. One to three additional trocars, including the AirSeal iFS (CONMED Japan KK, Tokyo, Japan), were placed to be used by assistant surgeons. To evaluate the tumor distribution and plan the excision margins, an ARIETTA 70 probe (Hitachi, Inc., Tokyo, Japan) was used for ultrasound examinations. After clamping the main renal arteries with the Bulldog on the affected side, the tumor was excised using cold scissors maintaining a secure margin of approximately 5 mm, and an inner running suture using 3–0 V-Loc (COVIDIEN Japan, Inc., Tokyo, Japan) was placed to repair the collecting system and large vessels. Following early unclamping of renal arteries, additional inner suturing and/or soft coagulation (VIO300D; ERBE Elektromedizin GmbH, Tübingen, Germany) was then carried out to stop the bleeding from interlobar or segmental arteries, followed by renorrhaphy using 2–0 V-Loc [22].

### Three-dimensional Volumetry

**Fig. 1:**
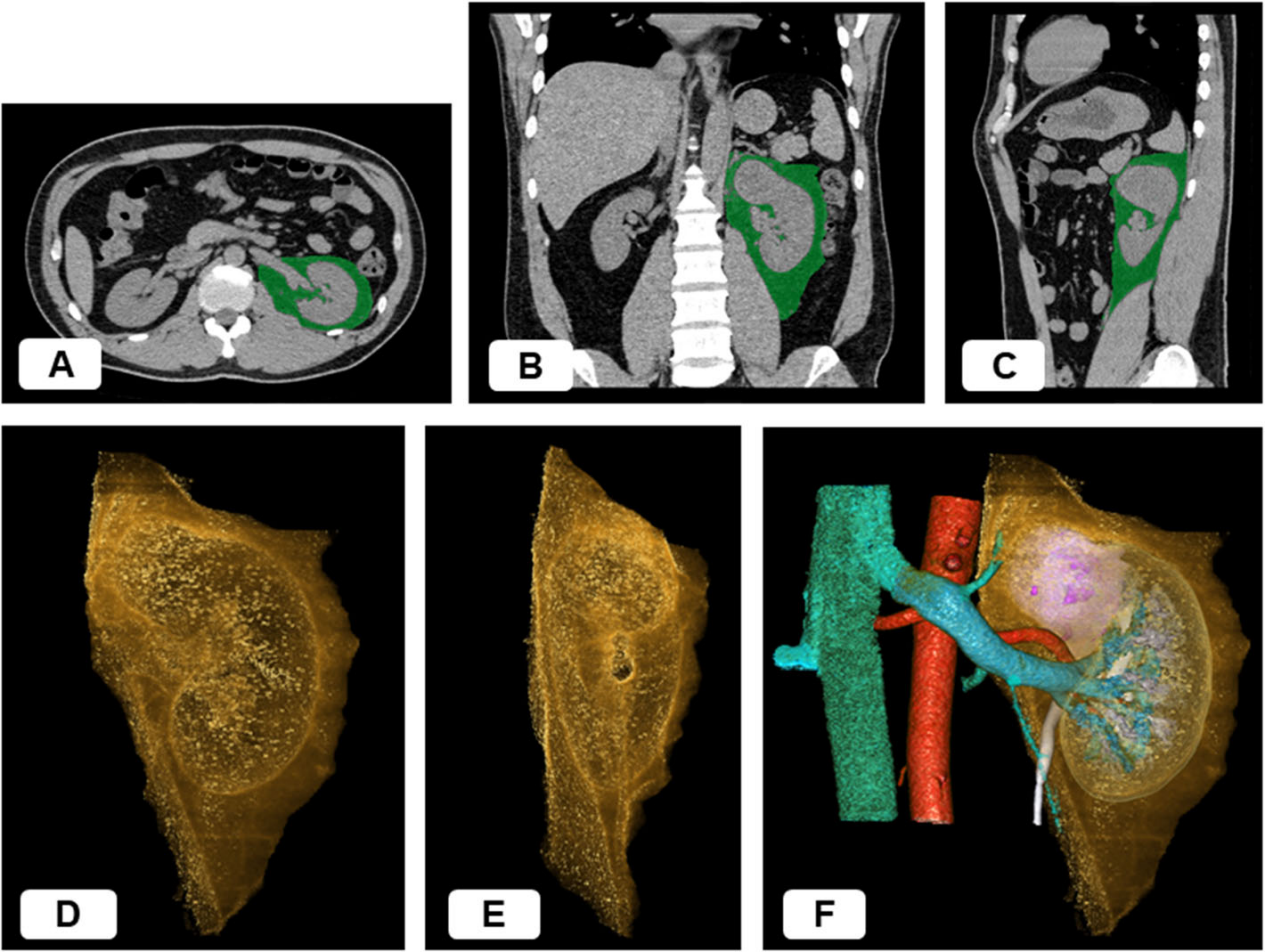
**a**, **b**, **c**: Preoperative non-contrast-enhanced computed tomography images for a patient with a localized renal mass located at the upper pole of the left kidney. The green area shows the region of interest corresponding to the perinephric fat defined as the fatty area within the anatomical structures, including the lateroconal fascia, fusion fascia, psoas muscle, lumbar quadrate muscle and diaphragm. **a**, Axial section. **b**, Coronal section. **c**, Sagittal section. **d**, **e**, **f**: The three-dimensional (3D) images for the perinephric fat reconstructed from the digital imaging and communications in medicine data according to the region of interest. The volume of this 3D structure was then calculated quantitatively. **d**, Anterior 3D view. The shape of the kidney and its upper tumor are stereoscopically excluded from the 3D structure. **e**, Inside 3D view. The lumens of renal hilar vessels are empty. **f**, Overlaid anterior 3D view showing the perinephric fat and other anatomical structures, including the kidney, artery, vein, ureter and tumor

For the volumetry of PNF, the region of interest (ROI) for PNF on the affected side was defined as the fatty area within the anatomical structures, including the lateroconal fascia, fusion fascia, psoas muscle, lumbar quadrate muscle and diaphragm. Based on the ROI, 3D images for PNF were reconstructed from the DICOM data of non-contrast-enhanced CT, and the volume of PNF was quantitatively calculated using the SYNAPSE VINCENT system (Fig. 1) [23].

### Statistical analysis

All statistical analyses were performed using R ver. 3.1.1 software (R Development Core Team, https://www.r-project.org/index.html), and *p*-values less than 0.05 were considered significant. Spearman’s rank correlation analysis was used to assess the association between the PNF volume and other parameters. Differences in the R.E.N.A.L. nephrometry score, PNF volume and console time during RAPN among the 5 groups consisting of 14 consecutive patients were assessed by the Kruskal-Wallis chi-squared test. Logistic regression analyses were conducted to examine the association between several parameters and the console time. Several variables with significant differences in the univariate analysis were selected and reanalyzed as the factors in the multivariate analysis.

## Results

The baseline clinical characteristics and perioperative outcomes of the 70 patients included in this study are summarized in Table 1. In this series, the median body mass index (BMI), MAP score and PNF volume on the affected side were 23.90 kg/m^2^ (ranged from 17.0 to 31.8 kg/m^2^), 0 (ranged from 0 to 4) and 166.05 cm^3^ (ranged from 22.1 to 1399.3 cm^3^), respectively, and the trifecta and MIC score system outcomes were achieved in 69 (98.6%) and 64 patients (91.4%), respectively.

**Table 1:** Patient characteristics and perioperative outcomes (*n* = 70)

Sex (%)	
Male	41 (58.6)
Female	29 (41.4)
Age (years), median (range)	63 (18–85)
Body mass index (kg/m^2^), median (range)	23.90 (17.0–31.8)
Mayo adhesive probability score, median (range)	0 (0–4)
Perinephric fat volume (cm^3^), median (range)	166.05 (22.1–1399.3)
Diabetes mellitus (%)	17 (24.3)
Hypertension (%)	29 (41.4)
Preoperative chronic kidney disease (%)	12 (17.1)
Tumor side (%)	
Right	38 (54.3)
Left	32 (45.7)
Hilar tumor (%)	15 (21.4)
Tumor size (mm), median (range)	23 (5–57)
R.E.N.A.L. nephrometry score, median (range)	7 (4–10)
Surgical approach (%)	
Transperitoneal	53 (75.7)
Retroperitoneal	17 (24.3)
Operative time (min), median (range)	167.5 (103–248)
Console time (min), median (range)	98.0 (60–194)
Estimated blood loss (ml), median (range)	50 (0–620)
Warm ischemia time (min), median (range)	12 (6–21)
Histological subtype (%)	
Clear cell renal cell carcinoma	46 (65.7)
Other malignant tumor	12 (17.1)
Angiomyolipoma	7 (10.0)
Other benign tumor	5 (7.1)
Positive surgical margins (%)	1 (1.4)
Major complications (Clavien-Dindo 3 or 4) (%)	0 (0)
Achievement of trifecta outcomes (%)	69 (98.6)
Achievement of MIC score system outcomes (%)	64 (91.4)

MIC, margin, ischemia and complications

**Fig. 2:**
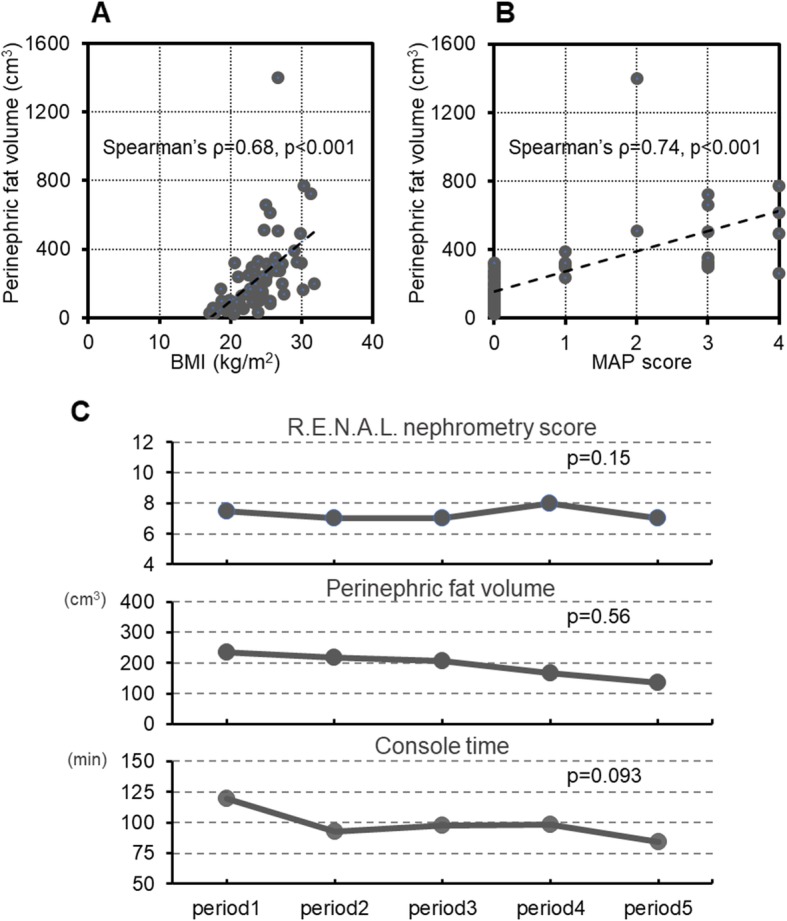
**a:** Assessment of the association between the perinephric fat volume and body mass index (BMI) by Spearman’s rank correlation analysis. **b:** Assessment of the association between the perinephric fat volume and Mayo adhesive probability (MAP) score by Spearman’s rank correlation analysis. **c:** Comparison of the median R.E.N.A.L. nephrometry score, perinephric fat volume and console time among the 5 groups consisting of 14 consecutive patients from the 70 most recent patients who underwent robot-assisted partial nephrectomy at our institution. Periods 1, 2, 3, 4 and 5 consisted of cases 1–14, 15–28, 29–42, 43–56 and 57–70, respectively

Scatter plots of the PNF volume according to the BMI and MAP score are presented in Fig. 2. The PNF volume was significantly correlated with both the BMI and MAP score in the 70 patients included in this study; however, the correlation coefficient was 0.68 between the PNF volume and BMI, whereas that between the PNF volume and MAP score was 0.74. The comparison of the R.E.N.A.L. nephrometry score, PNF volume and console time during RAPN among the 5 groups is also shown in Fig. 2. No significant difference was noted in the R.E.N.A.L. nephrometry score, PNF volume or console time among these 5 groups.

The outcomes of logistic regression analyses assessing the impacts of several parameters, including the PNF volume, on the console time as an index reflecting the complexity of RAPN are presented in Table 2. Univariate analysis identified the sex, MAP score and PNF volume as significant predictors of the console time. However, of these significant parameters, only the PNF was independently associated with the console time during RAPN by multivariate analysis.

**Table 2:** Univariate and multivariate analyses of several factors as predictors prolonging the console time during robot-assisted partial nephrectomy

	Univariate analysis^a^			Multivariate analysis ^a^		
	OR	95% CI	*p*-value	OR	95% CI	*p*-value
Age (< 65 versus ≥65 years)	0.71	0.28–1.84	0.49	–	–	–
Sex (male versus female)	3.47	1.27–9.50	0.015	1.29	0.39–4.32	0.68
Body mass index (< 22.0 versus ≥22.0 kg/m^2^)	0.52	0.18–1.53	0.23	–	–	–
Mayo adhesive probability score (0–2 versus 3–5)	0.07	0.01–0.57	0.013	0.18	0.02–1.67	0.13
Diabetes mellitus (positive versus negative)	3.32	0.93–13.85	0.052	–	–	–
R.E.N.A.L. nephrometry score (4–6 versus 7–12)	0.36	0.13–1.05	0.062	–	–	–
Hilar tumor (positive versus negative)	1.80	0.56–5.75	0.32	–	–	–
Surgical approach (transperitoneal versus retroperitoneal)	0.58	0.19–1.75	0.33	–	–	–
Surgical experience (less versus more than half)	2.00	0.77–5.18	0.15	–	–	–
Perinephric fat volume (< 166.05 versus ≥166.05 cm^3^)^b^	0.14	0.05–0.40	< 0.001	0.23	0.07–0.77	0.017

*OR* odds ratio, *CI* confidence interval

^a^The median value for console time (98.0 min) for the included patients was used as the cutoff point in this analysis

^b^The median value for perinephric fat volume (166.05 cm^3^) for the included patients was used as the cutoff point in this analysis

Variance inflation factors ranged from 1.10 to 1.25 in the present multivariate model

## Discussion

Currently available classification systems to estimate the complexity of PN are based on only tumor-specific factors, such as size and location, without taking patient-specific factors into account [8–10]. There have been a number of studies demonstrating obesity, particularly increased visceral fat, to be a potential patient-specific factor associated with surgical difficulty and frequent postoperative complications in renal surgery, including PN [11–14]. To date, several parameters reflecting obesity, such as the BMI, PNF thickness and APF, have been evaluated as predictors of the complexity of PN [11–17]; however, which parameter has the most significant impact on the complexity of PN remains controversial. Furthermore, the complexity of PN may be considerably influenced by the introduction of the robotic technique to this surgical field [2–6]. Taken together, the significance of obesity should be reanalyzed using more objective parameters to predict the surgical difficulty of PN in the era of robotic surgery. In this study, we hypothesized that the exact PNF volume can serve as a better predictor for the complexity of RAPN; therefore, this study was conducted to characterize the effects of the PNF volume on the perioperative outcomes in a total of 70 consecutive patients with clinical T1 renal tumors who underwent RAPN performed by a single experienced robotic surgeon beyond the initial learning curve.

In this study, we measured the PNF volume based on reconstructed 3D images using the SYNAPSE VINCENT system, which is a widely employed method to measure the volumetry of a specific target that has been reported to be able to calculate the exact volume with high reproducibility and interobserver concordance [23, 24]. The median PNF volume in the 70 patients included in this series was 166.05 cm^3^, but it ranged between 22.1 and 1399.3 cm^3^, suggesting its diverse distribution. We found a significant association between the PNF volume and other related parameters, such as the BMI and MAP score; however, these correlations were not close. Accordingly, it may be difficult to use either the BMI or MAP score as an alternative to the PNF volume measured by the method in this study.

Considering the effects of surgical experience on perioperative outcomes of RAPN, this study included the latest 70 patients undergoing RAPN at our institution during the period of this study. Furthermore, we confirmed the absence of a significant difference in the R.E.N.A.L. nephrometry score and PNF volume among the 5 groups. Based on these findings, this series consisted of patients with comparatively homogeneous features, and no significant difference in the console time during RAPN was noted. Therefore, the data from this cohort of patients may be sufficient to assess the impact of the PNF volume on the perioperative outcomes.

In this series, as achievement of the trifecta and MIC score system outcomes in the 70 patients was favorable, the components consisting of these systems, including the surgical margin status, ischemia time and postoperative complications, were not used as surrogates of the difficulty of RAPN. Therefore, we regarded the console time during RAPN as an index reflecting the complexity of RAPN, and assessed the effects of several parameters, including the PNF volume, on the console time. Of these, only the PNF volume was independently associated with the console time during RAPN. Our findings suggest that measurement of the PNF volume, rather than the BMI or MAP score, can help predict the level of difficulty of RAPN.

It is of interest to explore the etiology of why the console time during RAPN is independently affected by the PNF volume, irrespective of other factors, including the BMI or MAP score. BMI is a common factor to characterize the degree of obesity, but it reflects total body fat composition, and patients with the same BMI may have varying distributions of PNF. Indeed, several studies reported that the parameters associated with the distribution of intraabdominal fat are better predictors for PN outcomes than BMI [11, 25]. Furthermore, although it was originally developed to assess the probability of APF during PN [15], the MAP score, which is based on the posterior PNF thickness and stranding, was found to be significantly related to prolongation of the console time, particularly that of the dissection phase time, during RAPN [17]. However, the reproducibility of the MAP score was reported to be unsatisfactory [16], and the presence of APF itself may not be always affect the difficulty of PN [15]. Considering these findings, the volume of PNF, which markedly affects all surgical steps throughout the procedure of RAPN, is more likely to be associated with the complexity of RAPN than the BMI or MAP score.

There are several limitations in this study. First, as this was a retrospective study including a small number of patients, it will be necessary to confirm the findings presented in this study in a prospective study with a larger sample size. Second, RAPN for all 70 patients included in this series was performed by a single experienced robotic surgeon to minimize the effects of surgical experience on the study outcomes; therefore, the present findings cannot be applied to all cohorts of patients undergoing RAPN. Third, the procedure to exactly measure the PNF volume used in this study is slightly complicated; however, we believe that most urologists will be able to measure the PNF volume in approximately 10 min after a few procedures. Fourth, the volume of renal sinus fat, which usually has little impact on the RAPN procedure, was included in the measurement of the 3D volume because it is difficult to define the anatomical boundary line between perinephric and renal sinus fat. On the other hand, the definition of PNF in this series did not include the flank pad, although it may affect the complexity of RAPN, particularly when performed via the retroperitoneal approach. Last, although it is a standard value in our country, the BMI range in this series was lower than those in previous studies from the United States and Europe [15, 16]. Thus, it may be difficult to apply the present findings to cohorts with the significantly greater BMI.

## Conclusion

This study included a total of 70 patients with clinical T1a-b renal tumors who were treated by RAPN performed by a single experienced robotic surgeon beyond the initial learning curve, and the significance of the PNF volume as a predictive factor for the complexity of RAPN was analyzed. Considering the favorable trifecta and MIC score system outcomes, this study regarded the console time during RAPN as a surrogate for the index reflecting the surgical difficulty of RAPN, and only the PNF volume was found to be independently associated with the console time, irrespective other significant parameters, including the MAP score. Collectively, our study suggests that measurement of the PNF volume can serve as a reliable predictor of the complexity of RAPN if performed by an experienced robotic surgeon beyond the initial learning curve. To further investigate the significance of the PNF volume during RAPN, it will be of interest to assess the impact of the PNF volume on other surrogate points reflecting the surgical complexity in more diverse cohorts.

## Data Availability

All data generated or analyzed during this study are included in this published article.

## Abbreviations

3D: 3-dimentional
APF: Adherent perinephric fat
BMI: Body mass index
C-index: Centrality index
CT: Computed tomography
DICOM: Digital imaging and communications in medicine
MAP score: Mayo adhesive probability score
MIC: Margin, ischemia and complications
PN: Partial nephrectomy
PNF: Perinephric fat
RAPN: Robot-assisted partial nephrectomy
ROI: Region of interest
